# A Cross-View Geo-Localization Algorithm Using UAV Image and Satellite Image

**DOI:** 10.3390/s24123719

**Published:** 2024-06-07

**Authors:** Jiqi Fan, Enhui Zheng, Yufei He, Jianxing Yang

**Affiliations:** School of Mechanical and Electrical Engineering, China Jiliang University, Hangzhou 310018, China

**Keywords:** geo-localization, UAV, satellite, transformer, style transfer

## Abstract

Within research on the cross-view geolocation of UAVs, differences in image sources and interference from similar scenes pose huge challenges. Inspired by multimodal machine learning, in this paper, we design a single-stream pyramid transformer network (SSPT). The backbone of the model uses the self-attention mechanism to enrich its own internal features in the early stage and uses the cross-attention mechanism in the later stage to refine and interact with different features to eliminate irrelevant interference. In addition, in the post-processing part of the model, a header module is designed for upsampling to generate heat maps, and a Gaussian weight window is designed to assign label weights to make the model converge better. Together, these methods improve the positioning accuracy of UAV images in satellite images. Finally, we also use style transfer technology to simulate various environmental changes in order to expand the experimental data, further proving the environmental adaptability and robustness of the method. The final experimental results show that our method yields significant performance improvement: The relative distance score (RDS) of the SSPT-384 model on the benchmark UL14 dataset is significantly improved from 76.25% to 84.40%, while the meter-level accuracy (MA) of 3 m, 5 m, and 20 m is increased by 12%, 12%, and 10%, respectively. For the SSPT-256 model, the RDS has been increased to 82.21%, and the meter-level accuracy (MA) of 3 m, 5 m, and 20 m has increased by 5%, 5%, and 7%, respectively. It still shows strong robustness on the extended thermal infrared (TIR), nighttime, and rainy day datasets.

## 1. Introduction

Unmanned aerial vehicle (UAV) technology is heavily reliant on global navigation satellite systems (GNSS) for precise navigation and positioning; however, in practical applications, the stability and reliability of GNSS signals encounter multiple challenges. These challenges encompass signal blockage, external interference, and intentional signal spoofing, all of which can lead to service disruptions or diminished positioning accuracy. Consequently, the exploration and implementation of auxiliary or alternative positioning technologies beyond GNSS are vital to ensuring the reliable navigation of UAVs in complex environments. Presently, the primary candidate technologies include Inertial Navigation System (INS) [1,2], simultaneous localization and mapping (SLAM) [3,4], as well as vision-based positioning methodologies [5,6]. While INS can supply continuous positional information, they accumulate significant errors over extended operation periods. On the other hand, while SLAM techniques are potent, their effectiveness is constrained by the necessity for real-time mapping and a dependence on prior environmental knowledge. In recent years, with the rapid development of deep learning in the field of image processing, especially in terms of its excellent performance in tasks such as image retrieval [7], target detection [8,9], and tracking [10,11], the use of deep learning technology to process visual images and thereby achieve UAV positioning has become an integral part of positioning research. This approach not only surmounts certain limitations inherent in conventional methods but also harnesses abundant image features and sophisticated scene comprehension capabilities, thereby enhancing the robustness and precision of localization.

**Figure 1:**
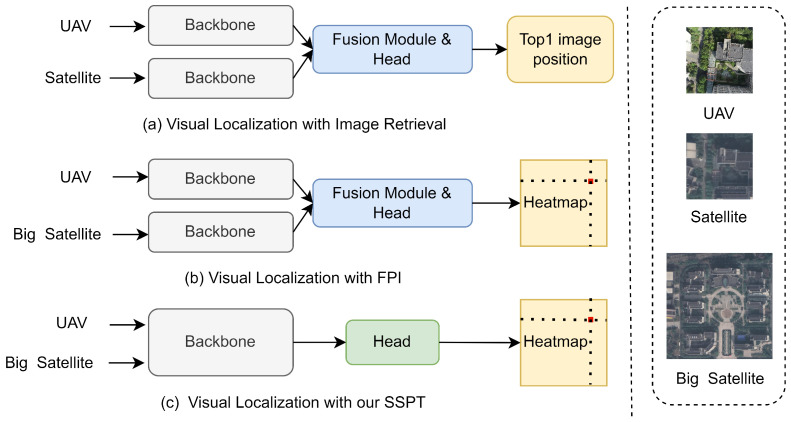
Comparison of visual localization frameworks. (**a**) A framework that realizes localization by retrieving the top-1 ranked image using image retrieval techniques. (**b**) A localization framework employing a dual-stream network followed by the fusion of resultant images for enhanced positioning. (**c**) Our proposed framework utilizes an SSPT network for efficient and accurate visual localization.

Within the current research landscape of unmanned aerial vehicle (UAV) visual positioning techniques, prevailing strategies can be broadly categorized into two classes: retrieval-based methods [12,13,14] exemplified by Figure 1a and the find point with image (FPI) [15,16,17] method based on the UL14 dataset, as depicted in Figure 1b. The image retrieval approach relies on constructing a vast image repository in which each image is tagged with precise positional information. During localization, the real-time image captured by the drone is matched with images in this library to estimate its position. However, this method not only necessitates the prior collection of a substantial volume of images within a specific area but is also susceptible to matching failures due to variations in viewing angles or minor environmental discrepancies, thereby impacting localization accuracy. In contrast, the FPI method directly localizes drone images onto large-scale satellite imagery, thus reducing reliance on an extensive image database. Nevertheless, it currently has limitations in terms of adaptable scenarios and requires improvement in terms of positioning precision. Both methodologies generally employ a backbone network to extract features from both drone and satellite images, followed by intricate fusion of these features and the application of head structures to correlate their respective feature spaces. This process, however, leads to a significant increase in computational cost, particularly due to the complexity of the head structure, which acts as a bottleneck in operational efficiency. Existing methods prove inadequate when dealing with visually similar yet geographically distinct scenes. We argue that this stems from insufficient interaction during the feature encoding stage, causing valuable information to be lost in the process. Specifically, during late-stage feature fusion, subtle differences that carry crucial localization cues tend to be diluted, thereby compromising the effectiveness of the overall system.

To address these challenges, we propose the SSPT network, illustrated in Figure 1c, which is designed to optimize network architecture and facilitate efficient early-stage feature integration, concurrently enhancing overall computational efficiency. Our study introduces a novel dynamic feature network rooted in attention mechanisms, aiming to facilitate effective interaction and sharing of image features right from the onset of training. The implementation of this network integrates both self-attention [18] and cross-attention [19] mechanisms, thereby dynamically adapting and optimizing feature extraction. This dual attention system within the model’s design allows for the concurrent training of drone and satellite images within the backbone network, promoting early synergy between their features. Self-attention enriches the depth and breadth of feature representations, while cross-attention effectively filters out irrelevant features, ensuring a focused concentration on critical information pertinent to the localization task. This design strategy significantly enhances localization accuracy. It is noteworthy that, while, to some extent, early feature fusion demonstrates significant performance enhancements compared to scenarios featuring non-interactive early-stage features, an overzealous pursuit of deep feature integration during initial stages has been observed to potentially undermine localization performance. This underscores the necessity for striking a delicate balance in the timing and degree of feature fusion.

In this task, the conventional simple upsampling methods, due to the constraints on the final output dimensions, often fail to accurately restore fine details, thereby introducing significant prediction errors in the resultant heatmaps. These errors directly impact the precision of localization. Consequently, our team, building upon the corner pyramid structure of Mixformer [20], has designed an upsampling head architecture specifically optimized for efficient upsampling. This design aims at meticulously recovering lost information through multi-level feature fusion and progressive magnification. Moreover, in the formulation of loss labels, we incorporate Gaussian kernel window functions as auxiliary means. Leveraging their smoothing characteristics and emphasis on local features, we achieve fine calibration of positions, fundamentally enhancing both the accuracy and robustness of localization under complex scenarios.

By employing advanced style transfer techniques, we successfully simulated visually diverse effects and sensor input conditions. While retaining the core values of the original UL14 dataset, the newly integrated subset covers images captured under nighttime illumination, scenes in adverse weather conditions such as rain, and challenging thermal infrared (TIR) imaging modes. Experimental results on these datasets demonstrate the model’s adaptability under different conditions, weather types, and imaging technologies, improving its ability to accurately position in complex real-world environments.

The main contributions of this paper are summarized as follows:

- We propose a novel SSPT (single-stream pyramid transformer) network in which self-attention and cross-attention mechanisms serve as pivotal components for feature extraction and fusion. This design facilitates joint feature extraction and relational modeling at an early stage of the processing pipeline, thereby enhancing both the localization precision and efficiency;
- A multi-scenario, multi-modal data augmentation strategy has been devised for unmanned aerial vehicle (UAV) image datasets, significantly broadening the diversity and complexity of the training data. Experimental results demonstrate that the SSPT network architecture exhibits exceptional adaptability and robustness, confirming its broad applicability in practical scenarios;
- The head structure of the model was redesigned to use a Gaussian function as the label weighting window. These two innovations help the model to better align with the central region of the label during pre-training and also promote precise localization in complex visual environments.

## 2. Related Work

### 2.1. Cross-View Visual Localization Research

In the progression of cross-view geo-localization techniques, researchers customarily employ satellite images imbued with geographic coordinates, aligning them with drone or street view imagery to accomplish precise positioning. Early methodologies predominantly relied on conventional feature matching techniques, such as scale-invariant feature transform (SIFT), for preliminary image similarity searches. Subsequently, Lin et al. [21] drew inspiration from the triumphs of deep learning in facial recognition, constructing a paired dataset comprising 78,000 ground-to-aerial perspective images using public datasets, and introduced the Where-CNN model, treating ground-to-air geolocation as an image retrieval task. Hu et al. [22] proposed the CVM-Net architecture, integrating Siamese networks [23] with NetVLAD [24] to collectively tackle cross-view matching problems. Zhu et al. [25] searched for matching images within a geotagged aerial overview reference database to estimate the GPS location corresponding to a street view image. Vo et al. [26] generated a new ground-to-air image dataset and devised novel loss functions for experimentation. Zhai et al. [27] were the first to introduce panoramic images in their study, coupling ground panoramas with satellite images to establish the CVUSA dataset, containing 35,500 matched samples, significantly broadening data diversity and viewpoint coverage. Building upon this, Liu et al. [28] expanded upon the CVUSA dataset by incorporating spatial orientation information (orientation maps), dubbing this augmented dataset CVACT, which further augmented models’ spatial comprehension and localization precision. Zhu et al. [25] addressed the limitation of prior designs that focused solely on central region matches, reframing the problem and positing a more pragmatic hypothesis: Points of interest in query images can appear anywhere. Under this hypothesis, they not only performed image retrieval but also incorporated regression analysis for finer localization, establishing the VIGOR dataset tailored for high-precision cross-view geo-localization.

With the proliferation of drone technology, drone-based visual positioning tasks have garnered increasing attention. Sui et al. [29] leveraged road information when matching drone images with satellite images in order to tackle the challenge of matching aerial urban scenes. By harnessing U-Net networks [30] for semantic extraction, they augmented matching accuracy. In 2020, Niu Liang et al. [31] designed four distinct network architectures adapted to varying scenarios, achieving commendable experimental results when deployed on drones. Zeng et al. [32] in 2022 introduced a framework of peer learning and cross-diffusion (PLCD) utilizing oblique drone views as a bridge between ground and satellite images for matching. Zheng et al. [33] incorporated the drone perspective into geolocation, defined target positioning and navigation tasks under the drone view, and assembled the University-1652 dataset based on Google Maps, covering streets and cities. ResNet-50 [34] pre-trained on ImageNet [35] is used as the backbone, forcing different images to be mapped to a shared space through shared weights, and using image retrieval methods to complete the task. The LPN network [36] uses the partition feature extraction method and replaces the VGG16 [37] network with ResNet. DAI designed the feature segmentation and region alignment module (FSRA) [38], using Transformer to replace the network backbone. Both improvements produced performance enhancements on the University-1652 dataset. Zhu et al. [39] created the SUES-200 dataset to focus on UAV positioning and navigation in real-world environments by matching UAV images captured at different altitudes with satellite images. Niu et al. [31] In order to solve the problem of GPS spoofing attacks on UAVs, the SATUAV dataset was constructed and data enhanced through techniques such as rotation, dimming, and fog simulation. The DenseUAV dataset by DAI et al. [12]. marked the advent of the first dataset tailored for autonomous drone localization, employing real low-altitude drone images paired with corresponding satellite images, setting a new benchmark for drone positioning research. Subsequently, based on DenseUAV, an unmanned aerial vehicle (UAV) imagery dataset UL14 [15] was constructed. expanding the satellite image coverage area from DenseUAV and devising the FPI network for pinpointing drone image locations within extensive satellite imagery, propelled advancements in drone positioning technology.

In general, current positioning methods mainly involve image retrieval, which uses aggregation algorithms to extract global features to match similar images, and many excellent related datasets have emerged. However, this method is greatly affected by environmental changes and has average positioning effect. The UL14 dataset produced by DAI et al. has changed the principle of using retrieval methods for positioning in the past, but the current “find points with images” method [15] requires a complex structure and a large amount of computing resources, and the accuracy level is low.

### 2.2. Vision Transformer

The fundamental innovation of the transformer architecture lies in its core component—the attention mechanism [22]—which was formally introduced by Google in 2017 to address problems related to sequential models. Departing from the use of convolutional neural networks (CNNs) [40,41,42] or recurrent neural networks (RNNs) [43,44,45], transformers enable high levels of parallel computation. Initially applied to natural language processing tasks [46,47,48] with remarkable success, it subsequently inspired Alexey et al. to propose the vision transformer (ViT) [49], ingeniously transforming image processing into a sequence problem by dividing images into patches, embedding positional information, and achieving excellent performance. Subsequently, other researchers successively launched the Swin transformer [50] with its multi-scale design, the pyramid vision transformer (PVT) [51,52] with a pyramid structure, and data-efficient image transformers (DeiT) [53] incorporating a distillation token for model performance optimization via knowledge distillation. Despite their individual distinctions, these exemplary networks all hinge on the attention mechanism, perpetually propelling the transformer architecture beyond its NLP origins.

In the attention mechanism, the process commences with generating three vectors: query (Q), key (K), and value (V). Subsequently, the relevance between query and key is computed, leading to a weighted aggregation of value to yield the attention-weighted output for each query. Both self-attention and cross-attention, as employed in this design, embrace the concept of shared query, key, and value, employing scaled dot-product attention for calculating attention weights. Post weight computation, both mechanisms apply these weights to derive outputs from the values. However, while self-attention operates with all information sourced from the same sequence, cross-attention diverges by having queries derived from one input sequence and keys and values derived from a distinct, separate input sequence, thereby facilitating inter-sequence information exchange. This provides conditions for the subsequent feature processing of drone images and satellite images.

### 2.3. Data Augmentation

The field of deep learning has long been plagued by a scarcity of dataset resources, spurring the development of numerous methods to augment datasets [54]. Initially, basic geometric transformations (such as rotation, scaling, shearing, and flipping) and photometric alterations (like changes in saturation, grayscale conversion, color jittering, etc.) were employed to expand datasets. Later on, techniques like MixUp [55], which involves pixel-wise blending of images, Cut-Mix [56] that replaces random areas with patches from other images, and Tokenmixup [57] emerged as variations of mixup strategies. These methodologies have proven effective in fundamental classification and detection tasks. In the study of cross-view geopositioning, in addition to the aforementioned methods, there has consistently been research on enhancements that simulate real-world environments. For instance, Niu et al. [9], addressing unmanned aerial vehicle (UAV) GPS spoofing attacks, constructed the SATUAV dataset, employing algorithms to introduce fog and grayscale adjustments, among other things. Nonetheless, these approaches mostly entail simple manipulations of the original images and cannot cater to specific augmentation needs dynamically.

The evolution of image style transfer technology [58,59,60,61] has offered robust support for addressing such issues. This technology empowers users to transcend the limitations of actual shooting environments. By providing a base content image and a reference style image they wish to emulate, a novel visual creation is generated, preserving the essential features of the content image while adopting the characteristics of the desired style. Image style transfer leverages neural network architectures to separately process and then combine content features (like object shapes and spatial layouts) and style features (color schemes, textures, and line patterns) through deep feature extraction, decoupling, and reconstruction. Notably, models represented by Pix2Pix [61] and CycleGAN [60] excel in this domain, achieving successful mappings from content to style images under supervised and unsupervised learning scenarios, respectively. In 2018, Maayan Frid-Adar and colleagues utilized GANs to enhance training datasets [62], significantly improving classification outcomes. Aysim Toker et al. [63] in 2021 proposed using adversarial generative networks to transform UAV imagery into ground street view images for geolocation purposes.

Lee et al. [64] leveraged the technique of image style transfer to generate realistic simulations of thermal infrared imagery (TIR), which effectively revealed temperature discrepancies on object surfaces. This type of imagery has widespread applications across multiple domains, such as military reconnaissance, medical diagnostics, and environmental protection. Similarly, this technique of image style transfer enables the creation of high-fidelity images replicating nighttime conditions and those captured under inclement weather circumstances. As a result, without incurring additional costs associated with field data acquisition, it significantly diversifies the sources of image data.

## 3. Methods

In this chapter, we will elaborate on the specific strategies behind the design and the setup details of the network architecture. In Section 3.1, we will outline the overall architecture of the network in detail, including the division of network layers, the interconnection methods between various modules, and the logical composition of the overall function. Section 3.2 discusses the selection process of the self-attention mechanism (SA) and cross-attention mechanism (CA) structures, as well as the design methods of patch embedding and position encoding. Section 3.3 dissects the feature pyramid upsampling structure within the prediction head, emphasizing its key role in enhancing feature resolution and spatial context understanding. Section 3.4 provides a simple comparison of different weight windows. The rationale for choosing a Gaussian window as the basis for assigning loss weights is further clarified. Finally, Section 3.5 investigates the style transfer techniques employed for dataset augmentation during experimentation, introducing the effects of the augmented datasets generated using these methods.

### 3.1. Network Overall Architecture

**Figure 2:**
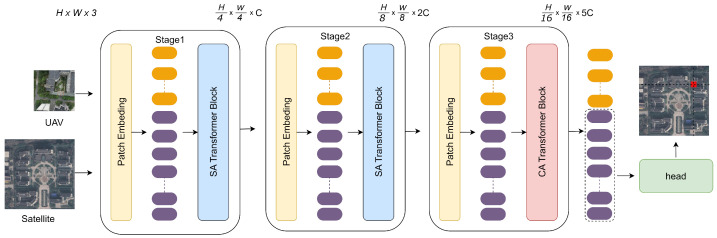
A network framework specifically designed for unmanned aerial vehicle (UAV) visual positioning. The backbone is built on the principle of comprehensive design, using the self-feature modeling layer and the cross-feature modeling layer as the core. The resulting feature output is post-processed by a dedicated head assembly to produce predicted positioning results overlaid on satellite imagery.

When building the network, we integrated advanced model design concepts and optimized the architecture based on the original FPI network to address the challenge of UAV image positioning. Unlike previous image retrieval techniques and find point with image-based visual positioning approaches, as shown in Figure 2, our framework initiates by feeding an image pair comprising a drone image (labeled Z, with dimensions $3\times H_{z}\times W_{z}$) and a satellite image (labeled X, with dimensions $3\times H_{x}\times W_{x}$), into the backbone network for feature extraction. The backbone network is referred to as SSPT and consists of three-level modules. Each successive stage halves the output resolutions H and W, while the number of channels C is set to 64, 128, and 320, respectively, resulting in specific outputs of spatial dimensions: $\frac{H}{4}\times \frac{W}{4}\times C$, $\frac{H}{8}\times \frac{W}{8}\times 2C$, and $\frac{H}{8}\times \frac{W}{8}\times 5C$, respectively. Each stage maintains a similar structure, commencing with a patch embedding layer that segments and maps the original 2D image matrices into a sequence of feature vectors amenable to transformer processing. These vectors then undergo profound transformation within each stage through a series of block components. At the heart of these blocks lie computational mechanisms split between self-attention and cross-attention. Self-attention focuses on uncovering intricate intra-image correlations, while cross-attention emphasizes extracting complementary information between the two images, synergistically enhancing the comprehensiveness and precision of feature representation. Finally, the resultant features are decoded and reconstructed by the head component, generating heatmaps imbued with precise positional information. This approach embodies a sophisticated and meticulously designed system, specifically configured to excel in localizing drone imagery with heightened accuracy and efficiency.

### 3.2. Transformer Based Backbone

Our SSPT network’s modeling module comprises two core components: patch embedding and block structures. The patch embedding method, inspired by the pvt_v2 network, employs an overlapping patch embedding technique to handle images, ensuring local continuity by utilizing overlapping block embeddings. To capture this continuity, patch windows are expanded to overlap neighboring regions partially, with zero-padding applied to maintain feature map resolution throughout the process.

The block units are primarily made up of layer normalization (LayerNorm), attention mechanisms, and a multi-layer perceptron (MLP) module augmented with a depthwise convolution (DWconv) layer. LayerNorm helps normalization during training, while 3 × 3 DWConv is introduced before GELU activation in MLP to act as a conditional position encoder and uses dropout’s regularization method to reduce overfitting.

**Figure 3:**
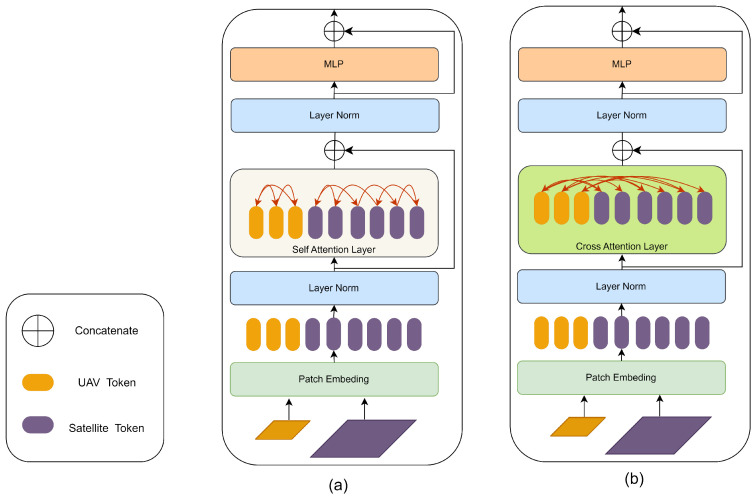
Layers constructed using various attention mechanisms (**a**) Self-attention feature modeling layer: Constituting an early-stage shallow model within the SSPT network, this layer primarily employs self-attention mechanisms to enhance intrinsic features of the data. (**b**) Cross-attention feature modeling layer: Integrated as a deep model in the latter stages of the SSPT network, this layer centers around cross-attention operations, which are fundamentally designed to facilitate inter-image feature interactions.

The feature modeling layers cleverly combines self-attention and cross-attention to enhance the ability of feature representation. The self-attention feature modeling layer is shown in Figure 3a. Self-attention is used to delve deeply into and aggregate the intrinsic features in a single image. The self-attention mechanism enables each position in the sequence associated with all other positions in the sequence. It calculates the similarity weight between different positions and the weighted summation in order to obtain the context representation of the current position. Given its configuration with a small number of stacked layers and the relative disorder of the initial input features, this module avoids introducing information exchange between images. This allows the model to focus on extracting and enhancing the underlying structure and features of a single image, thereby laying a solid foundation for subsequent feature integration. The cross-attention feature modeling layer is shown in Figure 3b, the modeling layer uses cross-attention to stimulate collaborative feature cooperation and information exchange between different image sources. The cross-attention mechanism involves the interaction between two different image sequences or modalities. In the encoder–decoder architecture, the cross-attention mechanism allows the decoder to dynamically focus on specific parts of the input sequence based on contextual information generated by the encoder. In this task, when the decoder captures the positioning target, it will focus on the relevant parts of the satellite image through the cross-attention mechanism to guide the positioning process. The key to cross-attention is that the keys and values come from one sequence, while the queries come from another sequence. This design allows the model to be used in different fine-grained dependencies between data sources.

These two attention mechanisms can be summarized by the Formula (1), which outlines the transformation of the input feature matrix Z from the drone image and the input feature matrix X from the satellite image into query (Q), key (K), and value (V). First, set X and Z to i; then, different linear mapping matrices—$W_{q}$, $W_{k}$, and $W_{v}$—are used to calculate with the input matrix i. Each element is converted into the corresponding Q, K, and V feature vectors.

(1) $$\begin{array}{cccc} \; & Q_{i}=iW_{q} & \; & i\in \{x,z\}\\ \; & K_{i}=iW_{k} & \; & i\in \{x,z\}\\ \; & V_{i}=iW_{v} & \; & i\in \{x,z\} \end{array}$$

Following the attention mechanism delineated in Equation (2), the process proceeds by calculating the similarity between each query vector and the ensemble of key vectors, subsequently undergoing normalization. The weighted summation of the corresponding value vectors, guided by these computed weights, produces the ultimate attention output. In the context of self-attention, the origins of the Q, K, and V vectors are unified within either the domain of X or Z. Conversely, with cross-attention, the query hails from the feature space of X or Z, while the key and value (K,V) vectors are from another feature space, inhabiting the distinct feature realms of X and Z, respectively. $d_{k}$, signifying the square root of the key vector’s dimensionality, serves a pivotal role as a scaling factor. This adjustment of attention weights fosters an environment conducive to numerical stability and refined optimization throughout the training lifecycle of the model.

(2) $$Attention\left(Q_{ij},K_{ij},V_{ij}\right)=\mathrm{softmax}\left(\frac{Q_{i}K_{j}^{T}}{\sqrt{d_{k}}}\right)V_{j}\;ij\in \left\{x,z\right\}$$

### 3.3. Pyramid Head

Following three successive stages of module processing, thorough interaction and integration of information between satellite and drone images is achieved. As depicted in the framework diagram, we initially present satellite and drone images as two independent sequences of inputs. Notably, only the processed sequence of satellite image features is ultimately an output, formatted as (B,5C,H/16,W/16), reflecting a relatively lower resolution. Direct upsampling of such low-scale features into heatmaps comparable in size to satellite images results in severe computational bias due to resolution differences.

**Figure 4:**
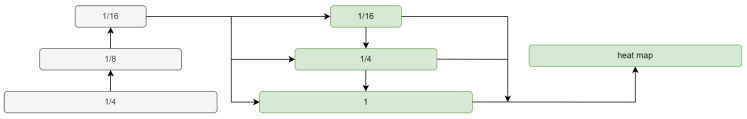
Pyramid head: The upsampled diverse feature vectors, enriched with multi-scale information, are fused through a pyramid head design. This integration strategy serves to minimize localization errors.

Given that the first two stages mainly perform separate self-attention calculations on their respective image sequences and that the last stage uses a cross-attention mechanism, the traditional feature pyramid network (FPN) architecture is not suitable for this situation. As illustrated in Figure 4, we adopt an innovative strategy: initially, upsampled features of the satellite images, formatted as (B,5C,H/16,W/16), are progressively upscaled to (B,1,H/4,W/4) and further to (B,1,H,W), thereby constructing a multi-scale feature pyramid. This pyramidal fusion of features enables comprehensive information integration across varying resolution levels, efficaciously mitigating the positional information loss and computational errors associated with direct upsampling. Consequently, the accuracy and reliability of the resultant heatmap predictions are significantly enhanced.

### 3.4. Loss Label Design

**Figure 5:**
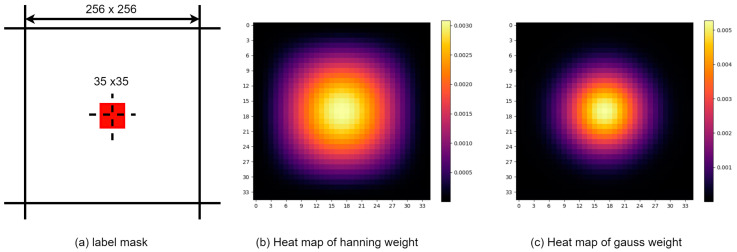
Overall label, and heatmap mask of positive samples for different weighted windows. (**a**) A label mask the same size as the satellite image. (**b**) Heatmap of weights produced by the Hanning window function. (**c**) Heatmap of weights resulting from the Gaussian window function.

During the training process, the coordinate locations in the satellite image corresponding to the drone image are initially assigned as label locations. Subsequently, a binary mask image is designed that is the same size as the satellite image, with a 35 × 35 square region centered at the label position being marked as positive samples, while the remaining areas serve as negative samples in the binary mask labels, as shown in the Figure 5a. Shown is the generated label mask. The purpose of choosing a square area of this size as the positive sample area is as follows: We assume that the original image will have a highest score point of 2 × 2 after downsampling 16 times, that it will be 32 × 32 after upsampling 16 times, and that there is a better transition from positive samples to negative samples. A threshold size of 3 is set for the transition sample area, and 35 × 35 is finally selected as the final positive sample area size. This can not only prevent the insufficient number of positive samples from causing difficulty in model convergence, but can also avoid misclassification of key information as negative samples during training. Finally, weights are assigned to both positive and negative samples in order to ensure finer-grained feature alignment. The hyperparameter sigma is used to adjust the weight assigned to positive samples, and the normalization method is used to ensure that the sum of the weights of positive samples and negative samples remains equal to prevent training data imbalance.

Positive sample weighting methods include average window function, Hamming window function, and Gaussian window function. The mean function distributes equal weights within its window, performing a simple uniform distribution on positive samples, but cannot distinguish the relative importance between them, resulting in the worst localization performance. In schemes such as FPI, the Hamming window function is used to set weights in a square area, as shown in Figure 5b, in order to guide the model to focus more accurately on the target center. The Hamming window function is a specific form of the raised cosine window; however, due to its relatively wide main lobe width, it is less effective at accurately converging to the target center in the later stages of training. In contrast, as shown in Figure 5c, the Gaussian window function, utilizes its narrower main lobe can better guide the model towards the actual target position, thereby achieving superior localization accuracy and improving localization results.

However, the value of sigma of the Gaussian function cannot be set too small. Although the smaller the sigma, the narrower the main lobe, this will cause the difference between the center weight and the edge weight of the positive sample to be too large, resulting in unnatural distortion in the interface area between positive and negative samples. At the same time, too small a sigma may cause the model to be too sensitive to noise or outliers in the training data, causing overfitting and weakening the model’s generalization ability. In the testing in this article, the best effect was found when sigma is set to 5.5.

In the design workflow, the adopted two-dimensional Gaussian window function is essentially an extrapolation of the one-dimensional Gaussian function across two spatial dimensions. The precise mathematical formulation of this function is given by Equation (3):

(3) $$G\left(x,y,\sigma \right)=\frac{1}{2\pi \sigma^{2}}\exp\left(-\frac{x^{2}+y^{2}}{2\sigma^{2}}\right)$$

Here, x and y correspond to the horizontal and vertical coordinate positions of pixels within the window, respectively, whereas σ signifies the standard deviation of the Gaussian distribution, which decisively influences both the spatial spread of the window and the falloff rate of its function values. Of particular note, under our application scenario, both positive and negative samples have a combined weight sum of unity; thus, we subsequently generate a normalized Gaussian window. As the training proceeds, we attentively fine-tune hyperparameters, including σ, based on practical circumstances, with the objective being to optimize weight assignments and overall model performance.

### 3.5. Dataset Augmentation

**Figure 6:**
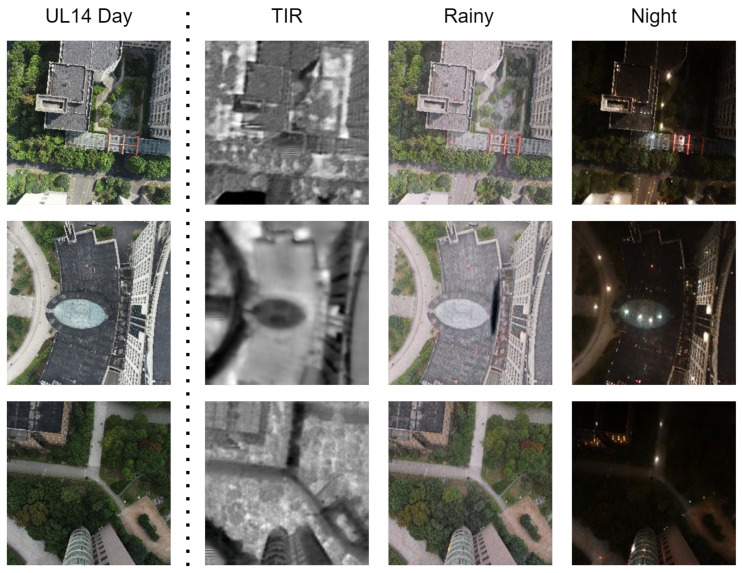
The effect of data generation: Diverse datasets were synthesized using the UL14 dataset, encompassing TIR (thermal infrared) format, rainy weather conditions, and nighttime scenarios, thereby significantly enriching the training environments.

The existing UL14 dataset primarily consists of standard images captured by cameras under clear weather and favorable lighting conditions. Acknowledging the diversity and complexity inherent in real-world applications, which encompass a multitude of factors such as varying climates, illumination conditions, and sensor configurations, we intentionally simulated and generated an array of drone image datasets across different environments, as illustrated in Figure 6. We refer to the original dataset as Day, and the expanded datasets are respectively named TIR, Rainy, and Night. For the synthesis of nighttime and rainy weather images, we employed the CycleGAN (cyclic consistency adversarial networks) approach to perform image to image translation. This method effectively transfers the style to images embodying nocturnal characteristics while preserving the original structural content. In the case of thermal infrared (TIR) image synthesis, we utilized a technique known as edge-guided multi-domain RGB-to-TIR image translation. This method leverages edge information from RGB images to guide the generation of corresponding TIR images, adeptly addressing the challenge of cross-modal image transformation. Both of these generative strategies represent efficacious and fruitful methods within the realm of style transfer. By leveraging pre-trained model weights, we swiftly tailored a substantial volume of realistic training data for specific tasks, thereby dramatically enhancing the drone’s recognition and adaptation capabilities in various challenging environments.

## 4. Experiment

### 4.1. Dataset and Evaluation Metrics

We performed testing and evaluation of our model using the UL14 dataset. The UL14 dataset comprises training samples consisting of pairs of carefully aligned drone and satellite images. Drone images are of the dimensions 512 × 512 × 3, and were captured at altitudes of 80 m, 90 m, and 100 m with an interval of 20 m between captures. On the other hand, satellite images are of the dimensions 1280 × 1280 × 3, and were cropped from Google Maps. Each pair of drone and satellite images is meticulously geo-spatially center-aligned, ensuring high precision in the subsequent creation of ground-truth labels.

Our dataset adds TIR, Rainy, and Night datasets to the original UL14 dataset. The image data statistics are shown in Table 1. In the training set, the ratio of drone images and newly generated data to satellite images is 1:1, with 6768 drone images (about 600 images per university) collected from 10 university areas and 6768 segmented satellite images. In the test set, the ratio of drone images and newly generated data to satellite images is 1:12, where each drone image corresponds to 12 satellite images of different scales at the same location (the actual area covered by the satellite image ranges from 180 m to 463 m), that is, 2331 drone images and 27,927 satellite images in 4 university areas, which can test the robustness of the model under multi-scale images.

In the testing phase, each drone image was paired with its corresponding position across 12 distinct satellite images, thereby constructing diverse testing scenarios. Furthermore, in the three newly constructed derivative datasets, namely UL14-TIR, UL14-Rainy, and UL14-Night, the original satellite image formats were retained, but different environmental conditions were simulated for the drone imagery, including thermal infrared imaging (TIR), rainy weather, and nighttime scenes. To ensure a rigorous comparison with existing literature and guarantee the reliability and comparability of our experimental results, we adopted evaluation metrics mentioned in the FPI paper. These metrics consist of the satellite image pixel deviation relative distance score (RDS) and the true geographical location deviation meter-level accuracy (MA). RDS is a measure quantifying the offset distance between the predicted peak position in the heatmap and the actual label position. The Formula (4) is given as follows, where dx and dy denote horizontal and vertical errors between predicted and true coordinates, w and h represent the width and height of the heatmap, and k, a hyperparameter set to 10 in this study, normalizes the error and accounts for image size effects:

(4) $$RDS=e^{\frac{-k\cdot \sqrt{{\left(\frac{dx}{w}\right)}^{2}+{\left(\frac{dy}{h}\right)}^{2}}}{2}}$$

For the MA (meter-level accuracy), geographical positioning accuracy is assessed using longitude and latitude. This metric transforms the model-predicted pixel coordinates into geographic coordinates (longitude and latitude), subsequently computing the mean absolute error between the predicted and true geographical coordinates. MA represents the percentage of total samples in which the approximate straight-line distance between the predicted location and the actual label location is less than D meters, thereby enhancing the geographic intuitiveness and interpretability of the evaluation outcomes.

### 4.2. Implementation Details

In our experiments, we employed the PyTorch 1.10.1 platform and devised a backbone architecture comprising three distinct stages with a depth of 3, 4, and 10 layers, respectively, thereby establishing a layered framework for feature learning. The backbone of our model was pretrained on the extensive benchmark dataset ImageNet 1K, with the objective of extracting fundamental and potent feature representations. Considering the constraints imposed by computational resources, we systematically examined various combinations of batch size and input resolution to determine the most advantageous parameter configuration. The model executed seamlessly on NVIDIA 1080Ti hardware, configured with a batch size of 24. Within the SSPT-256 network architecture, the input dimensions of drone images fed into the backbone network were uniformly processed to 96 × 96 × 3, whereas the dimensions of satellite images were adjusted to 256 × 256 × 3, a design strategy aimed at optimizing information extraction from diverse image sources. In terms of optimization, we adopted the AdamW algorithm and carefully set the learning rate: The learning rate of the backbone network was fixed at 0.0003, while the learning rate of the remaining parameters was 0.00045, thus providing a robust overall learning process for the model. The rate plan used CosineAnnealingLR, where T_max was set to the number of epochs, eta_min was set to 5 × 10^−6^, CenterR was set to 35, binary cross-entropy was used to calculate the loss, and the smooth decay of the learning rate was coordinated within 100 training cycles. Furthermore, the sigma of the Gaussian kernel was carefully calibrated to 5.5, a setting that facilitates accurate discrimination and effective generalization within complex feature spaces.In subsequent actual deployments, we considered NVIDIA Jetson TX2 as the core computing module, which has higher computing performance and lower computing power consumption and can realize larger and more complex deep neural networks.

### 4.3. Main Results

**Figure 7:**
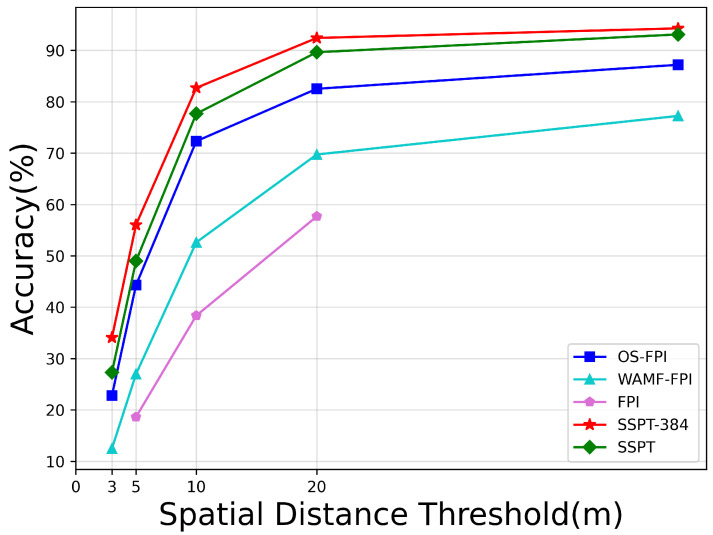
Performance comparison among different models based on the MA metric.

To ensure the rigor and comprehensiveness of comparative experimental outcomes, we conducted an in-depth evaluation on the UL14 dataset. As summarized in Table 2 and depicted in Figure 7, after standardizing input dimensions to 384 × 384 pixels for drone images and 96 × 96 pixels for satellite images, our experiments highlighted the exceptional performance of the SSPT-384 model. Notably, SSPT-384 surpassed all rival methods in the critical metrics of RDS and MA, with the SSPT-256 variant also demonstrating remarkable advancements in performance compared to other comparative approaches.

Specifically, SSPT-384 achieved an 8% increase over the previously best OSfpi model in the RDS metric. Under the MA criterion, for error thresholds at 3 m, 5 m, and 20 m, SSPT-384 enhanced location accuracy by 12%, 12%, and 10%, respectively. While SSPT-256 slightly trailed behind SSPT-384, it too made significant strides, improving upon OSfpi’s RDS by approximately 6% and exhibiting steady growth across multiple distance scales in the MA metric—boosting positioning performance by 5%, 5%, and 7% for errors within 3 m, 5 m, and 20 m.

It is noteworthy, however, that despite SSPT-384 outperforming SSPT-256 in both RDS and MA measures, the increase in satellite image dimensions by one-third necessitates more than double the computational resources. Due to the fact that high-computational-resource algorithms are unsuitable for real-world deployment scenarios in unmanned aerial vehicle (UAV) applications, and given that the performance gap between SSPT-384 and SSPT-256 falls within an acceptable range, we opted to proceed with all subsequent experiments on the SSPT-256 model, striking a balance between efficiency and practicality.

**Figure 8:**
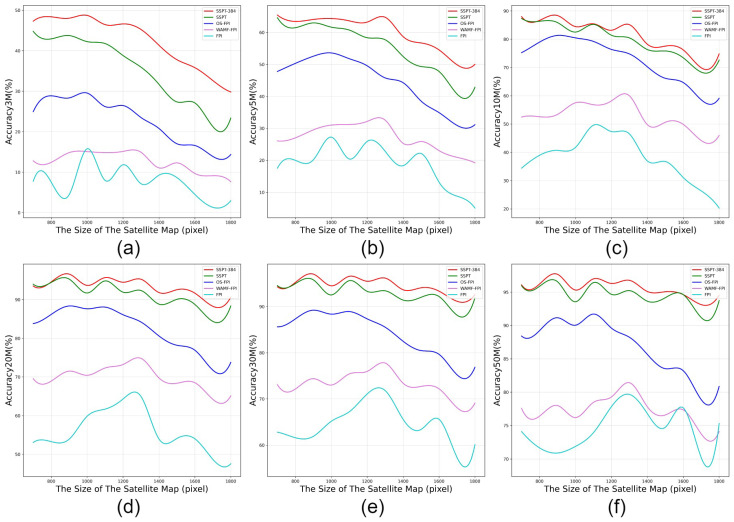
The performance of distinct models was systematically compared across different satellite map sizes. (**a**) Performance comparison of 3 m error. (**b**) Performance comparison of 5 m error. (**c**) Performance comparison of 10 m error. (**d**) Performance comparison of 20 m error. (**e**) Performance comparison of 30 m error. (**f**) Performance comparison of 5 m error.

As illustrated in Figure 8, the SSPT network exhibits a notably superior accuracy across various sizes of satellite map compared to other methodologies. Although there is a discernible decline in precision observed with the expansion of satellite map size within the 3-m error margin, the rate of accuracy decrement flattens out as we move into broader error ranges such as 5 m and 20 m and beyond. This stabilization in performance becomes increasingly evident with different map sizes. This phenomenon robustly validates the high resilience of the SSPT methodology when confronted with the challenge of recognizing satellite maps across multiple sizes.

### 4.4. Performance on the Other Dataset

As Table 3 illustrates, experiments conducted on the other dataset further substantiated the superior performance of the SSPT method. Even when confronted with diverse datasets including Thermal Infrared (TIR), Rainy, and Night, SSPT consistently excelled, achieving RDS accuracies of 76.72%, 80.09%, and 79.24%, respectively. Regarding the MA metric, for error thresholds of 3 m, 5 m, and 20 m, on the TIR dataset SSPT exhibited performances of 18%, 37%, and 82%, respectively, while on the Rainy and Night datasets, it maintained a high level of performance around approximately 22%, 42%, and 86%. This evidence suggests that, while variations in imaging formats as well as the introduction of lighting changes and weather conditions do have a certain impact on the final positioning accuracy, the SSPT method can always provide relatively reliable experimental results.

**Figure 9:**
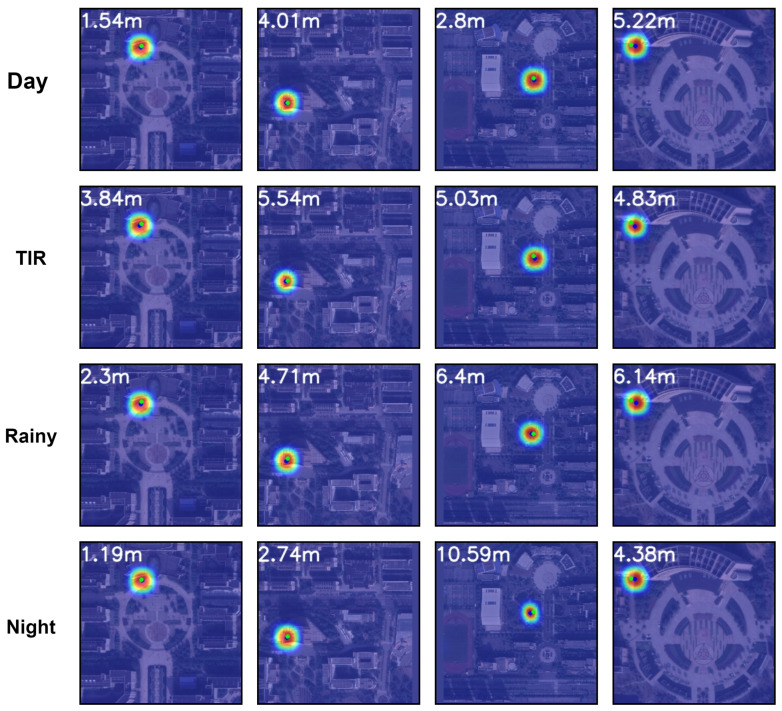
The heatmap effects of the SSPT model on partial images at different locations in different dataset.

Figure 9 presents intuitive heatmaps, which were generated from various types of images after processing through the network, overlayed onto satellite imagery. Green dots in the figure denote the positions of ground-truth labels, while the numerical values in the upper-left corner visually represent meter-level displacement distances between predicted results and actual labels, vividly demonstrating the spatial localization capability of the model.

## 5. Ablation Study and Analysis

In the ablation study, we will validate the impact of individual components on the model, including comparative experiments involving different numbers of cross-attention stages, experiments contrasting the use of pyramid structures for upsampling versus their absence, and experiments comparing the effects of employing different weight window allocation strategies.

### 5.1. The Effects of Cross-Attention and Self-Attention at Different Stages

**Figure 10:**
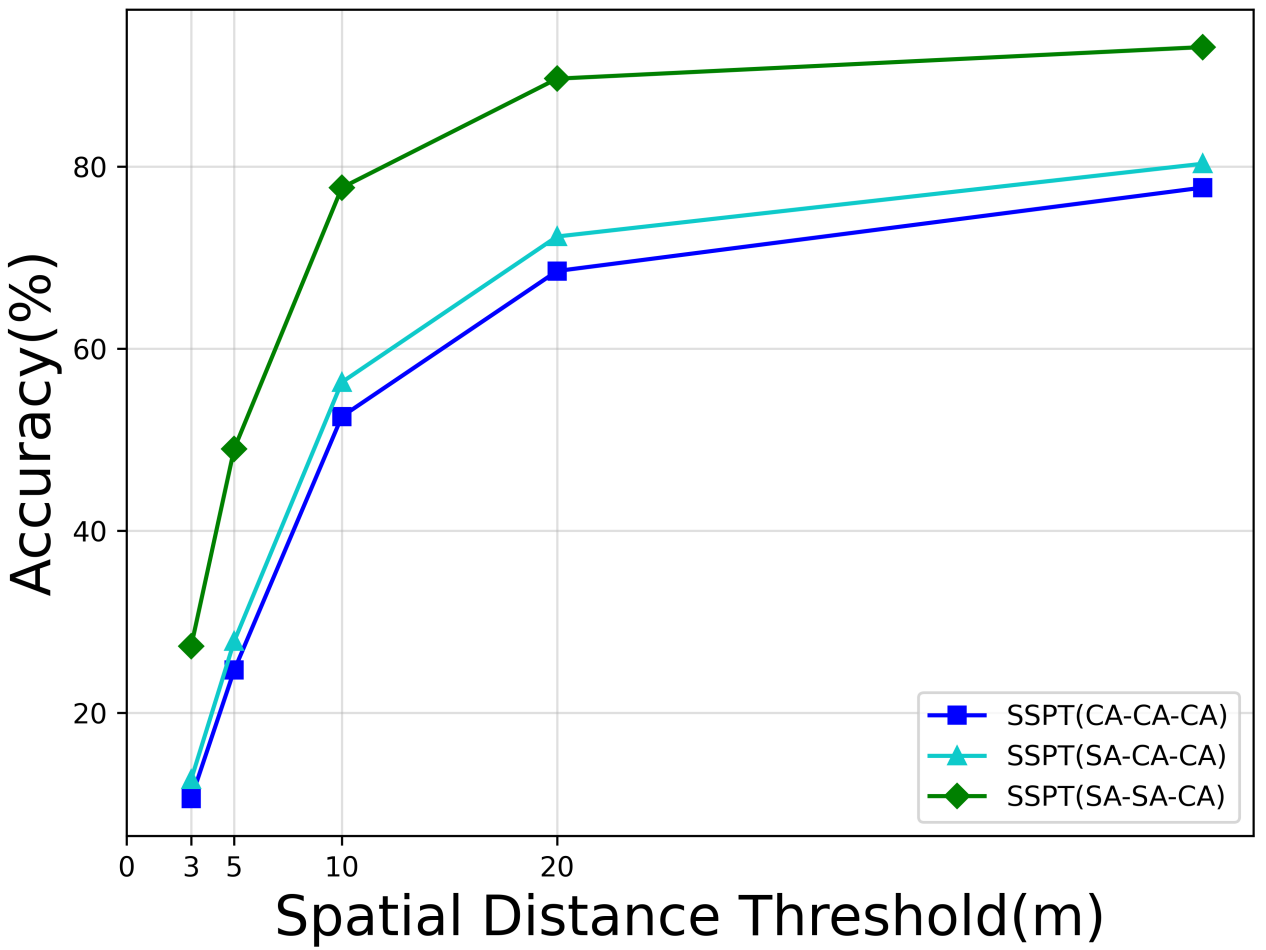
Performance comparison among varying numbers of cross-attention stages based on the MA metric.

As depicted in Figure 10 and Table 4, the variation in model performance is illustrated when integrating cross-attention mechanisms at different stage modules. The experimental setup comprises three distinct strategies: (i) all three stages adopt the cross-attention architecture (labeled as CA-CA-CA); (ii) self-attention is employed in the first stage followed by cross-attention in the subsequent two stages (designated as SA-CA-CA); and (iii) cross-attention is only incorporated in the final stage, with self-attention used in the preceding stages (notated as SA-SA-CA). The results indicate that the configuration in which cross attention is introduced solely in the last stage (SA-SA-CA) outperforms the other two configurations significantly.

The rationale for this phenomenon can be attributed to the inherent differences between drone and satellite images in terms of acquisition timing and technology, which render their content susceptible to substantial variations due to factors such as weather changes, lighting conditions, and seasonal shifts. Introducing cross-attention too early in the processing pipeline might lead to the model’s attention being dispersed among irrelevant or disruptive information during learning, thus compromising localization accuracy. By contrast, initially leveraging self-attention allows the model to focus on unique key features within each image, effectively filtering out distractions. Subsequently, combining cross-attention in deeper layers facilitates complementary fusion across modalities. This strategic approach better guides the model learning process, ultimately enhancing overall experimental outcomes.

### 5.2. The Effects of Pyramid Head

**Figure 11:**
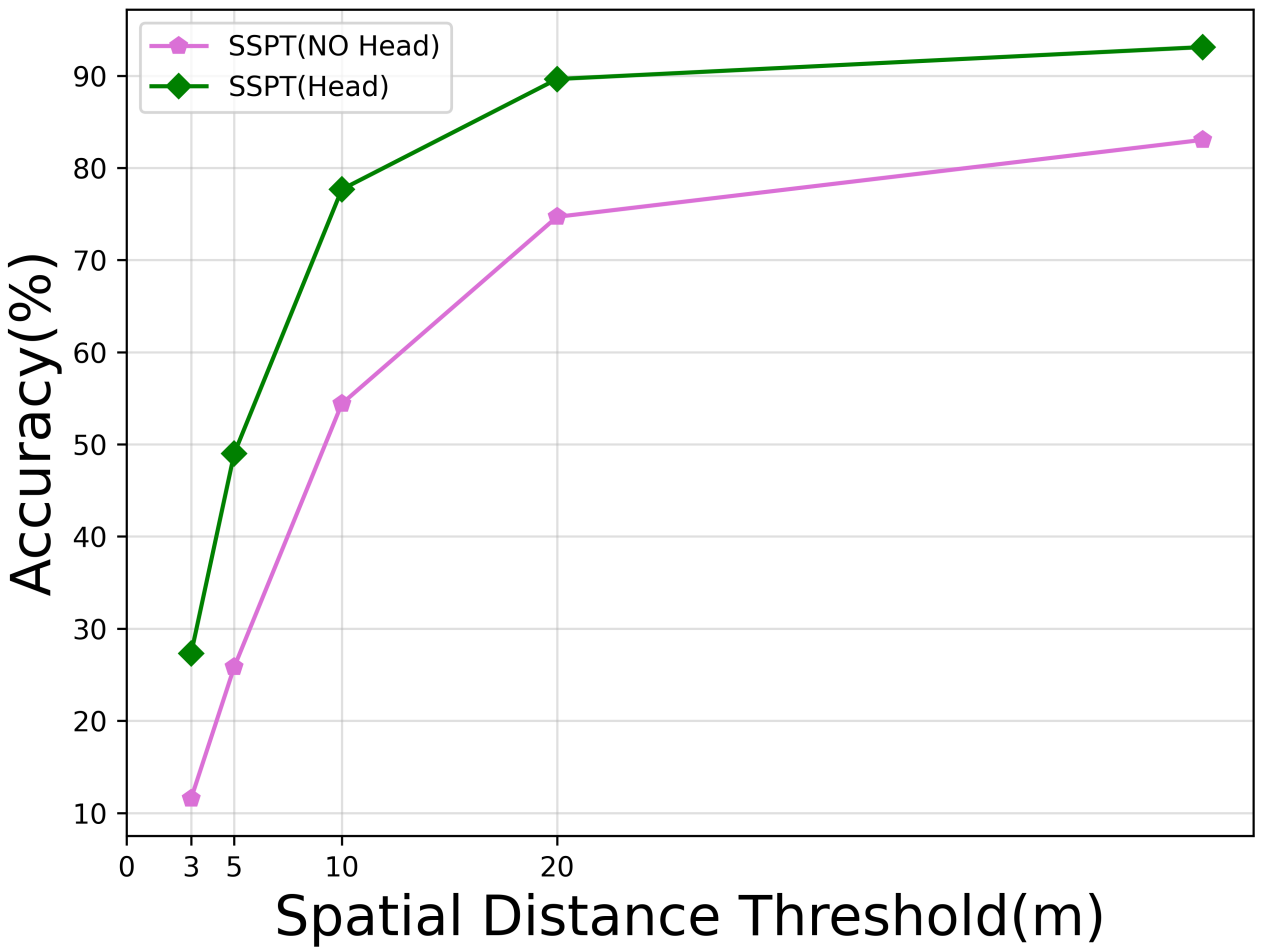
Performance comparison with and without pyramid heads based on the MA metric.

Due to the excessively low dimensions of the deepest feature vector space outputted by the backbone network, in order to generate heatmaps of the same dimensions as the satellite imagery, we adopted a strategy of upsampling these feature vectors. Despite an increase in computational load upon incorporating the head module, employing the pyramid head for upsampling yielded significant improvements compared to direct upsampling without a head. This is specifically illustrated through the results in Table 5 and Figure 11, where RDS improves by 12 percentage points, and performance within error ranges of 3 m, 5 m, and 20 m increased by 15%, 14%, and 23%, respectively. In fact, using the pyramid head lead to improved performance across all meter-level accuracy thresholds. The reason behind this lies in the fact that direct upsampling without the assistance of a head structure tends to result in losing spatial information, leading to considerable localization errors. Conversely, the pyramid head, with its multi-level and fine-grained information integration capabilities, can more accurately restore the original spatial details in the feature maps, thereby enhancing localization precision.

### 5.3. The Effects of Gaussian Window

**Figure 12:**
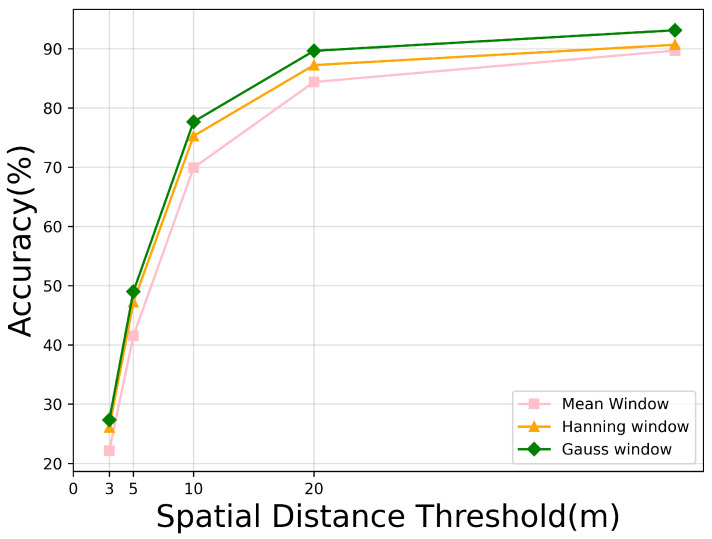
Performance comparison among different weighted window configurations based on the MA metric.

As Table 6 and Figure 12 reveal, the use of different window functions led to significant differences in experimental results. Of particular note is that the experiment outcomes were notably less favorable when employing the mean window strategy for weight allocation. The results indicate that its RDS value decreased compared with the other two window methods, and under the MA evaluation metric, even within smaller error margins, it did not perform as well as the other window methods. Only within larger error ranges did its performance approach that of the Hanning window. In contrast, while the Hanning window distinctly outperformed the mean window across various aspects, it still fell slightly short of the Gaussian window in terms of all performance indicators.

Delving into the basic principles, among these three window functions, when weights are distributed within an area of equal boundary range, the nature of the average window makes it impossible to accurately locate the center position of the positive sample; both the Gaussian window and the Hanning window can adjust the positive sample weights to focus the output on the core part of the main lobe of the function. Among them, the main lobe of the Gaussian window is narrow, which can locate the center of the positive sample better and faster in practical applications.

## 6. Discussion

In the exploration phase of drone cross-view localization technology, a critical challenge arises due to the significant differences between drone images and satellite images resulting from variations in imaging modalities, capture times, and illumination conditions. Hence, when employing the find point with image (FPI) method for cross-view positioning research, the selected localization algorithm must inherently possess exceptional robustness, superior adaptability, and efficient real-time response capabilities. The SSPT network architecture designed in this study provides a novel solution to this problem by integrating multiple attention mechanisms to build a joint feature model.

The thorough analysis of experimental data presented in Chapters Four and Five confirms that this proposed scheme has achieved substantial validation results not only on the original UL14 dataset but also on a variety of extended simulation environment datasets. Compared with other FPI-based methods, the SSPT network not only has good robustness when processing smaller-sized satellite images, but it also maintains high levels of positioning with varying meter-level accuracy in test scenarios involving satellite maps of different sizes. The excellent experimental performance of the SSPT-384 model convincingly highlights the enormous potential inherent in this network architecture.

## 7. Conclusions

In this research project, we have focused on cross-view geo-localization positioning technology for unmanned aerial vehicles (UAVs) and developed a novel network architecture coined SSPT. Through meticulous comparison with existing experimental results, our approach has achieved optimal performance across a range of evaluation metrics. Specifically, the backbone of the network incorporates meticulously designed stage modules that integrate both self-attention and cross-attention mechanisms aimed at efficiently extracting and fusing joint features and facilitating information exchange between images. Additionally, we introduce an innovative pyramid head structure that performs fine-grained, multi-scale upsampling on satellite image feature vectors. Moreover, we employ Gaussian functions to dynamically assign and optimize weights for positive sample windows.

In addition to algorithm enhancements, we also innovatively simulate real-life scenarios to enrich the dataset. However, the current work is limited by specific conditions, and the training is limited to UL14-related downward-facing UAV images in low-altitude urban environments. In future work, we will aim to expand the scope of our research, including exploring strategies for processing high-altitude drone imagery to cover a wider range of perspectives, collecting image data from remote and complex terrains, and applying algorithms to other relevant datasets. These efforts are expected to expand the adaptability and practicality of UAV visual positioning technology in different application environments.

## Figures and Tables

**Table 1:** Statistics of the UL14 dataset and newly generated data.

Dataset	Satellite	UAV	TIR	Rainy	Night	University
Train	6768	6768	6768	6768	6768	10
Test	27,972	2331	2331	2331	2331	4

**Table 2:** Comparison of the SSPT network with other methods on the public benchmark UL14.

Model	RDS	GFLOPS	Params
FPI	57.22%	14.88	44.48
WAMF-FPI	65.33%	13.32	48.5
OS-FPI	76.25%	14.28	14.76
SSPT-256	82.21%	7.23	21.47
SSPT-384	84.40%	15.28	21.47

**Table 3:** Performance evaluation of the SSPT network on generated datasets.

Dataset	RDS	<3 m (%)	<5 m (%)	<10 m (%)	<20 m (%)	<50 m (%)
UAV	82.21%	27.32%	49.00%	77.67%	89.64%	93.11%
TIR	76.72%	18.52%	37.43%	68.15%	82.78%	87.86%
Rainy	80.09%	22.40%	43.21%	73.05%	86.64%	90.51%
Night	79.24%	23.07%	42.54%	71.21%	86.06	90.45%

**Table 4:** Effects of RDS with varying numbers of cross-attention stages.

Stage	SSPT (CA-CA-CA)	SSPT (SA-CA-CA)	SSPT (SA-SA-CA)
RDS	66.18%	69.56%	82.21%

**Table 5:** Comparison of GLOPS and RDS with and without pyramid heads.

Pyramid Head	GLOPS	RDS
✕	6.39	70.12%
✓	7.23	82.21%

**Table 6:** Comparative study of RDS effects under different weighted window configurations.

Gauss Window	Hanning Window	Mean Window	RDS
✓			82.21%
	✓		79.19%
		✓	78.81%

## Data Availability

The datasets generated from the current study are available from the corresponding author on reasonable request.
